# Inhibiting HER3-Mediated Tumor Cell Growth with Affibody Molecules Engineered to Low Picomolar Affinity by Position-Directed Error-Prone PCR-Like Diversification

**DOI:** 10.1371/journal.pone.0062791

**Published:** 2013-05-10

**Authors:** Magdalena Malm, Nina Kronqvist, Hanna Lindberg, Lindvi Gudmundsdotter, Tarek Bass, Fredrik Y. Frejd, Ingmarie Höidén-Guthenberg, Zohreh Varasteh, Anna Orlova, Vladimir Tolmachev, Stefan Ståhl, John Löfblom

**Affiliations:** 1 Division of Protein Technology, School of Biotechnology, KTH Royal Institute of Technology, Stockholm, Sweden; 2 Affibody AB, Stockholm, Sweden; 3 Department of Medical Chemistry, Preclinical PET Platform, Uppsala University, Uppsala, Sweden; 4 Unit of Biomedical Radiations Sciences, Uppsala University, Uppsala, Sweden; Vanderbilt University, United States of America

## Abstract

The HER3 receptor is implicated in the progression of various cancers as well as in resistance to several currently used drugs, and is hence a potential target for development of new therapies. We have previously generated Affibody molecules that inhibit heregulin-induced signaling of the HER3 pathways. The aim of this study was to improve the affinity of the binders to hopefully increase receptor inhibition efficacy and enable a high receptor-mediated uptake in tumors. We explored a novel strategy for affinity maturation of Affibody molecules that is based on alanine scanning followed by design of library diversification to mimic the result from an error-prone PCR reaction, but with full control over mutated positions and thus less biases. Using bacterial surface display and flow-cytometric sorting of the maturation library, the affinity for HER3 was improved more than 30-fold down to 21 pM. The affinity is among the higher that has been reported for Affibody molecules and we believe that the maturation strategy should be generally applicable for improvement of affinity proteins. The new binders also demonstrated an improved thermal stability as well as complete refolding after denaturation. Moreover, inhibition of ligand-induced proliferation of HER3-positive breast cancer cells was improved more than two orders of magnitude compared to the previously best-performing clone. Radiolabeled Affibody molecules showed specific targeting of a number of HER3-positive cell lines *in vitro* as well as targeting of HER3 in *in vivo* mouse models and represent promising candidates for future development of targeted therapies and diagnostics.

## Introduction

The HER3 receptor is part of the epidermal growth factor receptor family, which is a group of tyrosine kinase receptors that mediate normal cellular functions such as proliferation and cell-to-cell interactions, but have also been recognized as drivers of many different human cancers [1]. Interestingly, HER3 differs from the other receptors of this family due to its inactive tyrosine kinase domain and hence, signals via ligand-induced heterodimer formation with other tyrosine kinase receptors [2], [3]. The HER2-HER3 signaling pair is an oncogenic unit in many HER2-driven breast cancers [4], [5], [6] and upregulation of the HER3 receptor has been shown to play a role in resistance to several currently approved drugs [7], [8], [9]. Moreover, the importance of HER3 in human cancers is not limited to HER2-driven breast cancers, and HER3 has also been shown to be involved in for example tumorigenicity of HER3-overexpressing prostate cancer xenografts *in vivo*, to maintain *in vivo* proliferation of a subset of ovarian cancers via an autocrine signaling loop, and to be involved in endocrine resistance of ER^+^ breast cancer cell lines [10], [11], [12]. Altogether, these findings demonstrate the potential of the HER3-signalling pathway as an important therapeutic target in human cancers and several anti-HER3 antibodies are currently under clinical investigations [13].

Recently, we have generated HER3-specific Affibody molecules of subnanomolar affinities, and demonstrated anti-proliferative attributes of these binders through blockage of ligand-induced HER3-signalling *in vitro* [14], [15]. These growth-inhibitory effects were demonstrated to be a result of competitive HER3-binding between the Affibody molecules and the ligand heregulin. The small and cysteine-free three-helical Affibody molecules are straightforward to further modify by conjugations and fusions, for example when developing multispecific targeting molecules [16], [17], [18]. In a recent study [19], Barbas and co-workers investigated this strategy using one of the HER3-specific Affibody molecules previously described by us [14], [15]. The Affibody molecule was fused with Cetuximab in order to generate a bispecific antibody targeting both EGFR and HER3. The new bispecific mAb/Affibody molecule gained a substantially improved *in vivo* efficacy in a xenograft tumor model compared to Cetuximab.

Altogether, these studies indicate that Affibody molecules might be used in future treatments of cancers that are dependent on heregulin-induced HER3 signaling. Ideally, the same targeting agent could be used for both therapy and diagnostics depending on the formatting. Affibody molecules have indeed been shown to also be excellent tracers for use in molecular imaging for diagnostic purposes [20] as they have the ability to accumulate at high levels in tumors due to high extravasation rate and high diffusivity [21]. However, for high-contrast molecular imaging of targets with relatively low expression (e.g. 10^4^ target proteins per cell), our recent results suggest that the affinity of Affibody molecules should be as high as possible, preferably in low picomolar range [22]. Molecular imaging of the HER3 receptor using unmodified Affibody molecules may thus be challenging due to the relatively low expression of the receptor on tumor cells (typically 10^3^ to 10^4^ per cell [23], [24]). Consequently, we wanted to further increase the affinity of the HER3-specific Affibody molecules prior to *in vivo* characterization. In addition, a higher affinity is generally favorable for improved therapeutic efficacy and might also enable bioactivity at lower drug doses, which would translate to longer effective duration of a therapeutic dose.

In this study, we present the generation of affinity-matured Affibody molecules targeting HER3. Initially, we performed an alanine scan of thirteen amino acid residues comprising the HER3-binding site of the previously best-performing Affibody molecule [14]. The alanine scan designated the importance of individual amino acid residues for HER3 binding and an affinity maturation library was designed based on these results. The library was displayed on *Staphylococcus carnosus* cells and fluorescence-activated cell sorting (FACS) of the library against human HER3 resulted in several Affibody candidates with significantly improved affinity towards the receptor. The two best candidates, denoted Z_08698_ and Z_08699_, demonstrated a substantially decreased dissociation rate compared to the original binder, with affinities in the low picomolar range. Encouraged by these results, *in vitro* cell binding of ^99m^Tc(CO)_3_-labelled Affibody molecules to a range of different cancer cell lines was analyzed, demonstrating receptor-specific binding of the Affibody molecules. Furthermore, biodistribution assays in mice were performed using ^99m^Tc(CO)_3_-labelled Z_08698_ and Z_08699_, showing receptor-specific uptake of the Affibody molecules *in vivo*. Finally, the new binders also demonstrated an improved inhibition of heregulin-induced receptor phosphorylation and more than two orders of magnitude lower IC50 value in a cell-based proliferation assay compared to the previously best-performing variant.

## Materials and Methods

### Labeling of HER3 and HSA

Biotinylation of recombinant human HER3-Fc chimera (R&D Systems, Minneapolis, MN) was performed using the Biotin-XX Microscale Protein Labeling Kit (Invitrogen, Carlsbad, CA) according to the supplier’s recommendations. The concentration of labeled protein was determined using amino acid analysis. Recombinant human HER3 (ECD, Sino Biological Inc., Beijing, China) was conjugated with biotin carboxylic acid, succinimidyl ester in NaHCO_3_ (0.1 M, pH 8.5) for 1.5 hours. Subsequently, glycine was added to stop the reaction followed by buffer exchange to PBS using a PD MiniTrap G-25 column (GE Healthcare, Uppsala, Sweden) according to manufacturer’s recommendations. Labeling of human serum albumin (HSA) with Alexa Fluor 647 succinimidyl ester (Invitrogen, Carlsbad, CA, USA) was performed according to supplier’s recommendations.

### Alanine Scanning Mutagenesis

Vector pSCZ_05416_ [14] was used as template for construction of the thirteen mutants. PCR amplification was performed with oligonucleotides encoding alanine replacements at residues 9, 10, 11, 13, 14, 17, 18, 24, 25, 27, 32 and 35 of the HER3-binding Affibody molecule Z_05416_ [14]. Amino acid A28 in the Affibody molecule was substituted to a valine by the same means. The PCR products were digested with *Nhe*I and *Xho*I restriction enzymes (New England Biolabs, Beverly, MA, USA) and ligated to the staphylococcal display vector pSCZ1 [25], digested with the same enzymes, using T4 DNA ligase (New England Biolabs) according to supplier’s recommendations. The *E. coli* strain RR1ΔM15 was used as host for plasmid construction and preparation was performed with a JETSTAR Kit (Genomed, Bad Oeynhausen, Germany) according to the supplier’s recommendations. BigDye Thermo Cycle Sequencing reactions and an ABI Prism 3700 instrument (Applied Biosystems, Foster City, CA) were used to verify the sequences. The constructs were transformed to electrocompetent *S. carnosus* TM300, according to previously described protocol [26].

### Flow-cytometric Analysis of Alanine Mutants

Staphylococcal cells displaying the respective mutants were inoculated to 10 ml tryptic soy broth supplemented with yeast extract (TSB+YE; Merck, Darmstadt, Germany) and 10 µg/ml chloramphenicol and grown overnight at 37°C and 150 rpm. 10^6^ cells from overnight cultures were washed with 800 µl phosphate-buffered saline supplemented with 0.1% Pluronic® F108 NF Surfactant (PBSP; pH 7.4; BASF Corporation, Mount Olive, NJ). The cells were pelleted by centrifugation (15000 *g*, 4°C, 6 min) and resuspended in 50 µl of PBSP containing 5 nM biotinylated HER3-Fc (R&D systems). The cells were incubated at room temperature for 2 hours with gentle mixing. The cells were washed with 180 µl ice-cold PBSP, followed by incubation in 200 µl ice-cold PBSP containing Streptavidin, Alexa Fluor 488 conjugate (Invitrogen, Carlsbad, CA) and 150 nM Alexa Fluor 647-HSA conjugate for 40 min in the dark. Following one wash with 180 µl ice-cold PBSP, cells were resuspended in 200 µl ice-cold PBSP prior to flow-cytometric analysis. The mean fluorescence intensity (MFI) was measured using an FACS Vantage SE (BD Biosciences, San Jose, CA) flow cytometer. The experiment was carried out in duplicate on different days using freshly prepared solutions.

### Grafting of Z05416 to an Optimized Affibody Scaffold

The gene sequence encoding the amino acids in positions 9, 10, 11, 13, 14, 17, 18, 24, 25, 27, 28, 32 and 35 of the HER3-binding Affibody molecule Z_05416_ was grafted to an optimized Affibody scaffold [27]. The gene sequence was digested with *Sac*I and *Xho*I restriction enzymes (New England Biolabs) and ligated to a staphylococcal display vector (digested with the same enzymes) using T4 DNA ligase (New England Biolabs) according to supplier’s recommendations. Plasmid construction and preparation, as well as transformation to electrocompetent *S. carnosus* TM300, was performed as described above.

### Library Design and Cloning

A SlonoMax library was obtained from Sloning BioTechnology GmbH (Pucheim, Germany), encoding helix 1 and 2 of the Affibody molecule with a *Xho*I and a *Sac*I site for subcloning to the vector (5′-CTC GAG GCG GAA GCC AAA TAC GCC AAA GAA XXX XXX XXX GCG XXX XXX GAG ATC XXX XXX TTA CCT AAC TTA ACC XXX XXX CAA XXX XXX GCC TTC ATC XXX AAA TTA XXX GAT GAC CCA AGC CAG AGC TCT-Ć; X denotes a randomized nucleotide position). The library was PCR amplified in 11 cycles using Phusion DNA polymerase (Finnzymes, Espoo, Finland) followed by purification of the PCR product using QIAquick PCR purification kit (Qiagen GmbH, Hilden, Germany). Subsequently, the library oligos were digested by *Xho*I and *Sac*I-HF restriction enzymes (New England Biolabs) and purified by preparative gel electrophoresis (2% agarose gel) using QIAquick gel extraction kit (Qiagen GmbH). A modified version of the *S. carnosus* expression vector pSCZ1 [28] was restricted by *Xho*I and *Sac*I-HF enzymes and purified by preparative gel electrophoresis as described above. The library oligos were ligated into the vector using T4 DNA ligase (New England Biolabs) at a 1∶5 molar ratio of vector to insert, followed by phenol-chloroform extraction and ethanol precipitation for purification and concentration of DNA fragments. Next, the library-encoding plasmids were transformed into electrocompetent *E. coli* SS320 by electroporation and individual clones where sequenced for library validation by BigDye Thermo Cycle Sequencing using an ABI Prism 3700 instrument (Applied Biosystems). The library plasmids were subsequently isolated using a Jetstar Maxi Kit (Genomed, Bad Oeynhausen, Germany), purified by phenol-chloroform extraction and concentrated by isopropanol precipitation. Finally, the library (hereafter denoted Sc:Z_HER3LIB2_) was transformed by electroporation into electrocompetent *S. carnosus* as previously described [26].

### Cell Labeling and FACS

Sc:Z_HER3LIB2_ was inoculated in TSB+YE supplemented with chloramphenicol (10 µg/ml) for overnight growth at 37°C and 150 rpm. The following day, cells were harvested by centrifugation (15000 *g*, 6 min, 4°C) and washed in PBSP before addition of biotinylated HER3. Cells were incubated with gentle mixing at room temperature prior to washing with ice-cold PBSP and labeling with Streptavidin, R-Phycoerythrin conjugate (SAPE; Invitrogen) as well as HSA conjugated with Alexa Fluor 647 for 30 minutes on ice at a concentration of 5 µg/ml and 300 nM, respectively. Cells were washed and resuspended in ice-cold PBSP. The library was labeled and sorted in four rounds using a MoFlo Astrios flow cytometer (Beckman Coulter, Indianapolis, IN). In sort 1 and 2, the library was labeled with 5 nM of biotinylated HER3. In sort 3 and 4, the library was subjected to off-rate selections. The library was first labeled with biotinylated HER3. After incubation and washing to remove unbound biotinylated HER3, the library was incubated with 5 nM of non-biotinylated HER3 (for 1 hour at room temperature in sort 3 and for 4 hours at room temperature in sort 4) prior to labeling with SAPE and HSA-Alexa Fluor 647. For each round of sorting, approximately ten times the library size was analyzed in the flow cytometer and the top fraction of cells (approximately 0.1–0.5%), with high HER3-binding to cell surface expression ratio, was gated and sorted into an eppendorf tube with TSB+YE. Subsequently, sorted cells were inoculated in TSB+YE with chloramphenicol (10 µg/ml) for overnight amplification prior to the next sorting round. Finally, isolated cells were spread on agar plates containing chloramphenicol and individual colonies were picked for BigDye Thermo Cycle Sequencing reactions using an ABI Prism 3700 instrument (Applied Biosystems, Foster City, CA).

### On-cell Screening for HER3 Binding

Individual staphylococcal clones from the sorting were inoculated in TSB+YE with chloramphenicol (10 µg/ml) and grown overnight at 37°C and 150 rpm. Cells were pelleted by centrifugation and washed in PBSP before resuspension in 0.5 nM and 2 nM of biotinylated HER3, respectively. After 1-hour incubation at room temperature with gentle mixing, cells were washed with ice-cold PBSP and labeled with SAPE at a concentration of 5 µg/ml and HSA-Alexa Fluor 647 at a concentration of 300 nM for 30 minutes on ice. Finally, cells were washed and resuspended in ice-cold PBSP. All samples were ranked based on the ratio between mean fluorescence intensities (MFI) from HER3-binding and cell surface expression signals in a Gallios flow cytometer (Beckman Coulter). In addition, the original HER3-binder Z_05417_ was analyzed for comparison.

### Expression and Purification of Soluble Affibody Molecules

Ten different HER3-binding Affibody molecules were produced and purified for further characterization. The Affibody-encoding DNA sequences were amplified from colonies by PCR, using primers that introduced *Nde*I and *Xho*I restriction sites. The Affibody sequences were subsequently inserted into the *Nde*I-*Xho*I restricted expression vector pET26b+ (Novagen, Merck KGaA, Darmstadt, Germany), generating monomeric Affibody constructs with a C-terminal His_6_-tag (H_6_). Rosetta (DE3) *E. coli* cells were transformed with the plasmids by standard heat-shock treatment. Cells were cultured in TSB+YE at 37°C and Affibody expression was induced by the addition of IPTG to a final concentration of 1 mM when the OD_600 nm_ had reached approximately 1. After incubation over night at 25°C, the cells were harvested by centrifugation at 6000 *g* for 8 minutes at 4°C. The cell pellets were resuspended in lysis buffer (7 M Guanidinium chloride, 47 mM Na_2_HPO_4_, 2.65 mM NaH_2_PO_4_, 10 mM Tris-HCl, 100 mM NaCl) and incubated for 2 hours at 37°C and 150 rpm. Subsequently, cell debris was removed by centrifugation at 4°C. The supernatant was isolated and Affibody molecules were purified by IMAC using a HisPur™ Cobalt resin (Thermo Scientific, Rockford, USA) under denaturing conditions. Buffer was exchanged to PBS by dialysis using Slide-A-Lyzer dialysis cassettes, 3.5 kDa cut-off (Thermo Scientific). The molecular weight and the purity of the purified Affibody molecules were verified by LC/MS (Agilent Technologies 6520 ESI-Q-TOF) and SDS-PAGE respectively. The protein concentration was determined by spectrophotometric measurement at 280 nm.

### Biosensor Off-rate Ranking

All biosensor assays were performed on a ProteOn XPR36 instrument (Bio-Rad Laboratories, CA, USA) using PBST as running buffer and 15 mM NaOH for regeneration. In all experiments, subtraction of responses from each sample over a blank surface was performed to minimize buffer contributions. Recombinant human and murine HER3-Fc (R&D systems) respectively, was immobilized on a GLC chip (Bio-Rad Laboratories) using standard sulfo-NHS/EDAC amine coupling chemistry. The ligands were diluted to a final concentration of 10 µg/ml in NaAc (10 mM, pH 4.5) and final immobilization levels were approximately 3000 RU, respectively. The purified Affibody molecules were injected at a concentration of 25 nM over the immobilized ligands at a flow rate of 100 µl/min. Association and dissociation time was 120 and 1800 seconds, respectively.

### Biosensor Analysis of HER3-binding before and after Heat Treatment

Affibody molecules, at a concentration of 25 and 50 nM, were subjected to heat treatment at 90°C for 15 minutes. Binding to HER3 was evaluated by injection of 400 µl of each sample before and after heat treatment over immobilized human and murine HER3 at a flow rate of 100 µl/min.

### Circular Dichroism Spectroscopy

Affibody molecules (0.2 mg/ml) were subjected to circular dichroism at 195–250 nm and 20°C using a Jasco J-810 spectropolarimeter (Jasco Scandinavia AB, Mölndal, Sweden) in a cell with an optical path-length of 1 mm. In addition, the ellipticity at 221 nm was measured for each Affibody molecule while heating the sample from 20 to 90°C (5°C/min).

### Biosensor Affinity Determination

For determination of the equilibrium dissociation constant (K_D_), a GLC sensor chip (Bio-Rad Laboratories) was immobilized with human HER3-Fc (R&D systems) as described above, with a final immobilization level of approximately 650 RU. Duplicate injections of a two-fold dilution series of each Affibody molecule ranging from 1.8 to 7.2 nM for Z_08698_ and 1.7 to 6.8 nM for Z_08699_, was injected over immobilized human HER3 (protein concentrations were determined by amino acid analysis). The flow rate was set to 50 µl/min and the association and dissociation was followed for 300 seconds and 4 hours, respectively.

### Materials for *in vitro* and *in vivo* Assays

High-quality Milli-Q water (resistance higher than 18 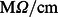) was used for preparing solutions. ^99m^Tc was obtained as pertechnetate from an Ultra-TechneKow generator (Covidien, Dublin, Ireland) by elution with sterile 0.9% NaCl. IsoLink labeling kits were kindly provided by Covidien. NAP-5 size exclusion columns were from GE Healthcare. Cells used during *in vitro* experiments were detached using trypsin-EDTA solution (0.25% trypsin, 0.02% EDTA in buffer, Biochrom AG, Berlin, Germany). Radioactivity was measured using an automated gamma-counter with a 3-inch NaI(Tl) detector (1480 WIZARD, Wallac Oy, Turku, Finland). The purity of radiolabeled Affibody molecules was determined by radio-ITLC (150–771 DARK GREEN, Tec-Control Chromatography strips from Biodex Medical Systems, New York, USA) and cross-validated by SDS PAGE. The distribution of radioactivity along the thin layer chromatography strips was measured on a Cyclone™ Storage Phosphor System and analyzed using the OptiQuant™ image analysis software (PerkinElmer). Data on cellular uptake and biodistribution were assessed by an unpaired, two-tailed *t*-test using GraphPad Prism (version 4.00 for Windows GraphPad Software, San Diego California USA) in order to determine significant differences (p<0.05).

### Labeling and *in vitro* Cell Binding of ^99m^Tc(CO)3-Z_08698_-H_6_ and ^99m^Tc(CO)3-Z_08699_-H_6_

Labeling of Z_08698_-H_6_ and -Z_08699_-H_6_ with [^99m^Tc(CO)_3_]^+^ was performed as described by Orlova and co-workers [29]. Briefly, 500 µl (200–320 MBq) of ^99m^TcO_4_^−^ -containing generator eluate were added to a vial with the IsoLink kit. The mixture was incubated at 100°C during 20 min. Thereafter, 40 µl of mixture were transferred to a tube containing 50 µg (∼6.8 nmol) Affibody molecule in 40 µl PBS and incubated at 50°C for 60 min. The labeling yield was measured by ITLC. When the ITLC strips are eluted with PBS, pertechnetate, as well as carbonyl and histidine complexes of ^99m^Tc migrate with the eluent front (R_f_ = 1.0), while Affibody molecules do not migrate under these conditions (R_f_ = 0.0). To determine the presence of reduced hydrolyzed technetium, ITLC was eluted with pyridine:acetic acid:water (5∶3:1.5). When this eluent is used, the technetium colloids stay at the application point (R_f_ = 0.0), while radiolabelled Affibody molecules, as well as pertechnetate and carbonyl complexes of ^99m^Tc migrate with the solvent front (R_f_ = 1.0). In addition, blank experiments were performed, where the Affibody molecules were omitted. The labeled Affibody molecules were purified using NAP-5 desalting columns, pre-equilibrated and eluted with PBS. The purity of each preparation was evaluated using ITLC.

The specificity of ^99m^Tc(CO)_3_-Z_08698_-H_6_ and ^99m^Tc(CO)_3_-Z_08699_-H_6_ for binding to HER3-expressing cells was evaluated using LS174T colorectal carcinoma, NCI-N87 gastric carcinoma, MCF-7 breast carcinoma, LNCaP and DU-145 prostate cancer cell lines (American Type Tissue Culture Collection, ATCC, via LGC Promochem, Borås, Sweden.) An *in vitro* specificity test was performed according to the methods described earlier [30]. Briefly, a solution of radiolabelled Affibody molecules (at 1 nM) was added to 6 Petri dishes (around 2×10^6^ cells in each). For blocking, 0.7 µM of non-labeled Affibody molecule was added 15 min before radiolabeled conjugates to saturate the receptors. The cells were incubated during one hour in a humidified incubator at 37°C. Thereafter, the media was collected, the cells were detached by trypsin-EDTA solution, and the radioactivity in cells and media was measured to enable calculation of the fraction of cell-bound radioactivity.

### *In vivo* Studies

All animal experiments were planned and performed in accordance with national legislation on laboratory animals’ protection and were approved by the Ethics Committee for Animal Research in Uppsala.

The goal of the biodistribution experiment in normal NMRI mice was to test specificity of *in vivo* accumulation of radiolabeled Affibody molecules in organs where HER3 is expressed (lung, liver, stomach, small intestines, salivary gland), as well as to study biodistribution of Affibody molecules. To evaluate if uptake in these organs was saturable, ^99m^Tc(CO)_3_-Z_08699_-H_6_ Affibody molecule (65 kBq in 100 µl PBS per mouse) was intravenously injected in a group of four female NMRI mice (average weight 24.5±1.6 g). The injected protein dose was adjusted by dilution with non-labeled Affibody molecule to 1 µg (ca. 0.13 nmol) or 10 µg (ca. 1.3 nmol) per mouse. At 4 h pi, four mice were sacrificed by injection of a lethal dose of anesthesia (20 µl of Ketalar-Rompun per gram body weight: Ketalar (50 mg/ml, Pfizer), 10 mg/ml; Rompun, (20 mg/ml, Bayer) followed by heart puncture and exsanguination with a syringe rinsed with heparin (5000 IE/ml, Leo Pharma). Samples of blood, lung, liver, spleen, stomach, small intestines, kidney, uterus, salivary gland, muscle and bone were collected, weighed and their radioactivity was measured. ^99m^Tc radioactivity was measured in the energy range of 100–160 keV. The data were corrected for background. Tissue uptake was calculated as percent of injected radioactivity per gram (% IA/g). Radioactivity in carcass was calculated as % IA per whole sample.

To evaluate targeting of HER3-expressing tumors *in vivo*, mice bearing prostate cancer xenografts were used. LNCaP cells (around 6×10^6^) were implanted in right hind leg of male BALB/C nu/nu mice in 50% Matrigel. The biodistribution experiments were performed 4 weeks after implantation, when tumor weight was 0.8±0.4 g. The average animal weight was 20.1±0.6 g at the time of experiment. To evaluate if uptake in these organs was saturable, ^99m^Tc(CO)_3_-Z_08698_-H_6_ and ^99m^Tc(CO)_3_-Z_08699_-H_6_ (85 kBq in 100 µl PBS per mouse) was intravenously injected in a group of three mice. The injected protein dose was adjusted by dilution with non-labeled Affibody molecule to 0.1 µg (0.013 nmol) or 10 µg (1.3 nmol) per mouse. The animals were sacrificed at 6 h after injection, and biodistribution was measured as described above.

### Inhibition of Cell Proliferation

The cell line MCF-7 was propagated as recommended in complete medium (RPMI 1640 medium with L-glut (Lonza, Basel, Switzerland)) supplemented with sodium pyruvate (Lonza), non-essential amino acids (Lonza), penicillin/streptomycin (Lonza) +10% fetal calf serum (FCS) (Gibco, Invitrogen). At the day of experiment, cells were washed twice in RPMI 1640 without supplements and resuspended in assay medium (RPMI 1640 medium with L-glut containing sodium pyruvate, non-essential amino acids, penicillin/streptomycin, +2% dialyzed FCS (Gibco)). The ability of the Affibody molecules (Z_08698_, Z_08699_ and Z_05417_) to block heregulin-induced proliferation was analyzed by mixing the binders with 200 pM heregulin (NRG1-β1/heregulin1-β1 EGF domain, R&D Systems) in assay medium. The molecules were titrated in a 5-fold dilution series with a fixed concentration of heregulin (200 pM). Furthermore, in an additional dilution series for each Affibody molecule, 9 µM recombinant human serum albumin (HSA, Novozymes, Bagsvaerd, Denmark) was added to the assay medium. The titration was performed in 96-well cell culture plates in a volume of 100 µl. 1500 cells were added per well (100 µl) and plates were incubated at 37°C, 5% CO_2_ for five days. After incubation, determination of the number of living cells in each well was performed using cell counting kit-8 (CCK-8, Fluka, Sigma Aldrich). 19 µl of CCK-8 cell proliferation reagent diluted two-fold in RPMI 1640 medium was added per well and absorbance was measured after 4 hours at 450 nm using a microplate reader (Victor3, Perkin Elmer). The data on cell growth was assessed by non-linear regression, and the half maximal inhibitory concentration (IC50) was determined using GraphPad Prism (GraphPad Software, San Diego California USA).

### Inhibition of Receptor Phosphorylation

Inhibition of receptor phosphorylation was essentially performed as described earlier [15] with a fixed concentration of heregulin (4 nM) and Affibody molecule concentration of 40 and 400 nM.

## Results

### Alanine Scanning

**Figure 1:**
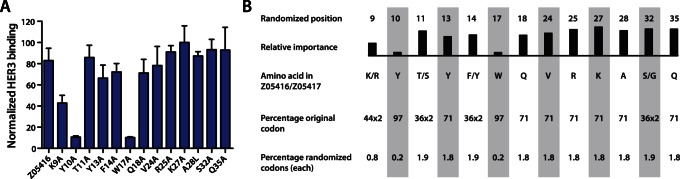
Alanine scanning and library design. A. Alanine scanning of Z_05416_. Histogram showing the results from flow-cytometric analysis of the thirteen staphylococcal-displayed mutants. The residues in the Affibody molecule that were mutated are represented on the x-axis, and a ratio of the mean fluorescence intensity (MFI) corresponding to HER3 binding and the MFI corresponding to surface expression level (monitored by HSA binding) is represented on the y-axis. B. Library design. Schematic overview showing the randomized positions in helix 1 and 2, the relative importance of the respective residue for HER3 binding (as determined by alanine scanning), the amino acids in each position in the original HER3 binders, the percentage of the codon corresponding to the original amino acid and the percentage of each of 15 or 16 (depending on the position) mutation codons.

Alanine scanning mutagenesis was used to determine the contributions from individual amino acids of an Affibody molecule in binding to the extracellular domain of HER3. The thirteen originally randomized residues in the Z-scaffold were substituted to alanine (except for position 28 that was mutated from alanine to valine), respectively, and each construct was subcloned into a staphylococcal display vector for subsequent transformation and expression on the staphylococcal host. Thirteen clones of staphylococcal cells, each displaying one of the thirteen mutants, were incubated with labelled HER3-Fc and the relative impact of each mutation on HER3-binding was analyzed using flow cytometry. Variants with alanine mutations at positions 9, 10, and 17 demonstrated substantially lower signals compared to the control, indicating a reduced affinity for HER3 and that the corresponding original residues were contributing to the interaction (Figure 1A).

### Grafting of Z_05416_ to a New Affibody Scaffold

A second-generation Affibody scaffold with improved properties was recently described [27]. The optimized scaffold contains eleven amino acid substitutions in the non-binding surface and demonstrates an increased thermal and chemical stability, as well as more efficient chemical synthesis [27]. We sought to base our maturation library on this improved scaffold. However, prior to synthesizing the affinity maturation library, we wanted to confirm that the HER3-specific Affibody molecule was compatible with the eleven mutations in the framework. The selected sequence of the HER3-specific Affibody molecule (Z_05416_) was therefore grafted into the optimized scaffold and the new variant was subcloned to a staphylococcal display vector and expressed on staphylococci. Staphylococcal cells displaying the grafted Affibody variant as well as the original Affibody molecule (Z_05416_) were incubated with labeled HER3-Fc and analyzed using flow cytometry. The HER3-specific Affibody molecule that was grafted to the optimized Z-scaffold demonstrated a similar HER3-binding signal as well as cell surface expression as the binder in the old scaffold, thus indicating full compatibility (data not shown).

### Library Design

The affinity maturation library was based on the amino acid sequences of the parental Z_05416_ and Z_05417_ as well as the results from the alanine scanning. The intention of the library design was to mimic the mutagenesis output from an error-prone PCR amplification, but restrict the mutations to 13 surface-exposed residues on helix 1 and 2. In order to achieve this, each randomized position was spiked with the codon that was encoding the original amino acid in addition to a mix of mutation codons that was encoding the rest of the amino acids (except aspartic acid, cysteine and proline). We decided to aim at an average mutation frequency of three mutations per molecule. Since we targeted thirteen positions for randomization, the average proportion of mutation codons in each position was therefore 3/13 • 100 = 23%, and thus an average proportion of 77% of the original codon. The theoretical binomial distribution of mutation frequencies in the library is shown in Figure S1A. The production of the randomized DNA was performed using the Slonomics technology from former Sloning BioTechnology GmbH (now part of the MorphoSys Group), which is similar to trinucleotide synthesis and enabled the design of the randomizations without biases associated with degenerative codons. To potentially increase the functionality of the final library, the mutation frequency in each position was normalized with the corresponding alanine scanning data, i.e. a position that was demonstrated to be important for the interaction was mutated at a lower frequency and vice versa. The final library design is outlined in Figure 1B.

### Library Construction

**Figure 2:**
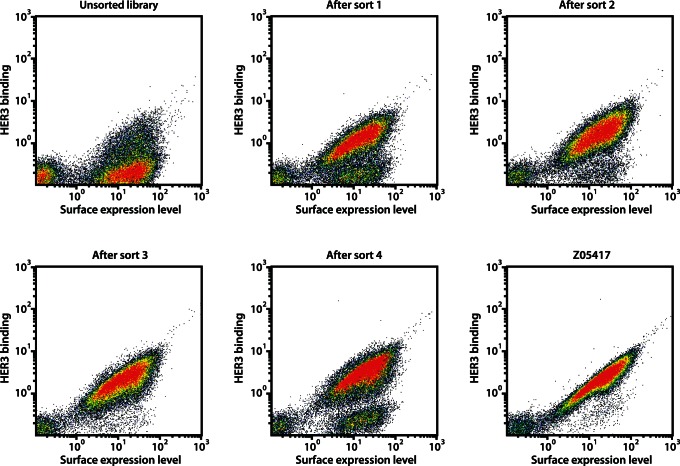
Fluorescence-activated cell sorting of Sc:Z_HER3LIB2_. Density dot plots of the affinity maturation library displayed on *S. carnosus*, showing the HER3-binding signal on the y-axis and the surface expression level on the x-axis. The dot plots show cells from the unsorted library as well as cells isolated from the first, second, third and fourth sorting round, respectively. For comparison of HER3-binding signals, a dot plot showing Z_05417_ is included.

The library was subcloned into a staphylococcal surface display vector and transformed into *S. carnosus,* yielding approximately 6.7 • 10^7^ individual clones. Theoretical calculations on library diversity, library coverage and combinatorics of the library design are shown in Figure S1B. The library was analyzed by DNA sequencing, which verified an amino acid distribution in acceptable accordance with the library design and a low percentage of frame shifts (1.8%, data now shown). However, the mutation frequency was somewhat lower than the theoretical design, with an average of approximately 2 mutations per clone (data not shown). Flow-cytometric analysis of Sc:Z_HER3LIB2_ revealed a high fraction of full-length library-expressing clones (around 80%), as shown by the HSA-binding signal, and demonstrated retained binding of a fraction of library clones to HER3 (Figure 2), which was expected due to the relatively moderate mutation frequency in the library.

### Flow-cytometric Sorting of Affinity Maturation Library

Fluorescence-activated cell sorting (FACS) is an efficient method for isolation of cells with desirable traits from combinatorial libraries. As we have shown previously, flow-cytometric sorting in combination with staphylococcal display enables identification of high-affinity binders towards many different targets of interest [14], [28]. In order to isolate Affibody molecules with high affinity towards HER3, Sc:Z_HER3LIB2_ was sorted in four rounds against biotinylated HER3. Detection of receptor binding in each sorting cycle was performed using Streptavidin-conjugated R-Phycoerythrin and the target-binding signal was normalized against the surface expression level through labeling of cells with HSA-Alexa Fluor 647 conjugate. In each sorting round, the top fraction of cells, showing high HER3 binding compared to surface expression level, was isolated and amplified by overnight growth prior to the next round of sorting. The selection stringency was modified throughout the selection process by changing the sorting parameters and gates, and also by decreasing the target concentration as well as incorporating off-rate selections in later sorting rounds. As shown by flow-cytometric analysis after each selection cycle, the sorting procedure resulted in successful enrichment of HER3-binding clones (Figure 2). In addition, enrichment of binders with improved HER3-binding signals compared to the original binder Z_05417_ could be observed throughout the sorting. After the third and the final sorting round, individual colonies were sequenced for identification, yielding 107 unique sequences out of 272 total reads. In summary, these results suggested a successful library design and selection of affinity-matured Affibody molecules targeting HER3.

### On-cell Affinity Ranking

After the sorting of Sc:Z_HER3LIB2_ by FACS, isolated clones were analyzed individually in order to identify Affibody molecules with the highest affinity towards HER3. Altogether 40 unique clones from both the third and the fourth sorting round were affinity-ranked by flow cytometry, by determining the ratio between the HER3 binding signal and the surface expression level (FL-2/FL-6). The vast majority of analyzed clones showed improved affinity towards HER3 compared to the original HER3-binder Z_05417_ (data not shown) and ten top candidates (hereafter denoted Z_08694_–Z_08703_) were subcloned and purified from *E. coli* cell extracts as C-terminally His_6_-tagged Affibody molecules.

### Biosensor Off-rate Ranking

**Figure 3:**
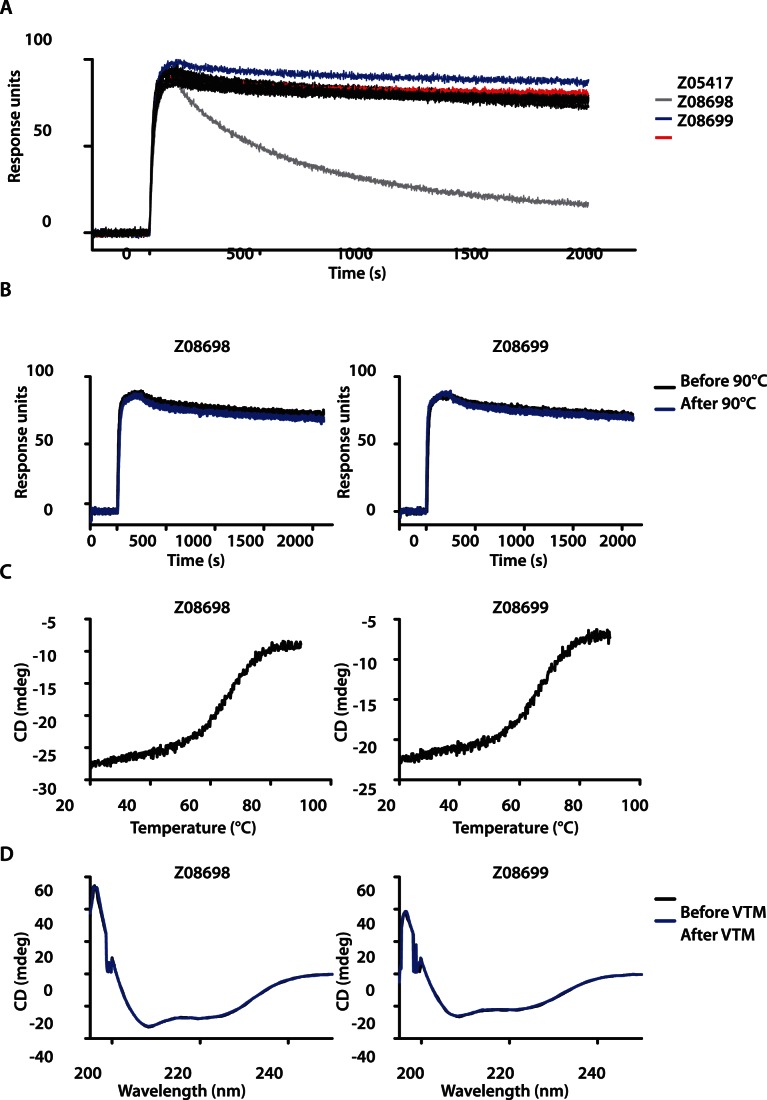
Characterization of affinity-matured Affibody molecules. A. Biosensor off-rate ranking of ten affinity-matured HER3-binders. Sensorgrams of ten different Affibody molecules injected over immobilized human HER3-Fc (all affinity-matured clones are shown in black, except for Z_08698_ and Z_08699_, which are shown in blue and red, respectively). For comparison, the HER3-binder Z_05417_ was included in the analysis (grey curve). B. Analysis of refolding capacity and thermal stability of affinity-matured HER3-binders evaluated by SPR and CD. Sensorgrams showing injections of 50 nM Z_08698_ and Z_08699_, respectively, before and after heat treatment at 90°C over immobilized human HER3-Fc. C. Variable temperature measurement (VTM) spectra obtained at 221 nm while heating the HER3-specific Affibody molecules Z_08698_ and Z_08699_ from 20 to 90°C. D. CD spectra of Z_08698_ and Z_08699_ at wavelengths ranging from 195 to 250 nm at 20°C.

All ten purified Affibody molecules were subjected to an off-rate ranking by biosensor analysis in order to further narrow down the number of HER3-binding variants to those demonstrating the highest affinity towards the receptor. All ten Affibody variants were injected over a sensor chip surface immobilized with human HER3-Fc and dissociation was followed during 30 minutes. All affinity-matured Affibody molecules generated similar binding curves to HER3 with significantly reduced off-rates compared to the original HER3-specific Affibody (Figure 3A). For further evaluation of the HER3-specific Affibody molecules, we focused on the two candidates, Z_08698_ and Z_08699_, which demonstrated the slowest off-rates.

### Biosensor Analysis of HER3-binding before and after Heat Treatment

Therapeutic effect of the HER3-specific Affibody molecules could potentially be mediated by the ability to block heregulin-induced tumor growth, but as an alternative, the small and stable Affibody molecules could easily be conjugated with different payloads, such as radionuclides. Radionuclide labeling is also employed when the binders are used as tracers in molecular imaging. Such labeling often requires heating of the protein to high temperatures, which motivated the evaluation of the refolding capacity of the affinity-matured Affibody molecules. Therefore, the interaction between HER3 and the Affibody molecules (Z_08698_ and Z_08699_) before and after heat treatment at 90°C was evaluated by SPR technology. The Affibody molecules, before and after heating, were injected over immobilized human and murine HER3-Fc for comparison of generated sensorgrams. As shown in Figure 3B, the sensorgrams from both Affibody molecules overlapped before and after heat treatment, demonstrating that the binding capacity of the Affibody molecules was unaffected by the thermal denaturation and refolding. The same was observed for binding to the murine HER3 receptor before and after heating (data not shown). These results were encouraging but not surprising due to the generally high solubility and efficient refolding of Affibody molecules [16].

### Circular Dichroism Spectroscopy

For further investigation of the thermal stability, the Affibody molecules were subjected to circular dichroism spectroscopy. The melting temperatures of both binders were determined by measuring the ellipticity at 221 nm while heating the proteins from 20 to 90°C. The obtained melting curves corresponded to T_m_ values of 64 and 65°C for Z_08698_ and Z_08699_, respectively (Figure 3C), demonstrating improved thermal stabilities compared to the original HER3-specific Affibody molecules. Additionally, spectra at wavelengths ranging from 195–250 nm were obtained before and after the variable temperature measurement in order to assess the reversibility of the unfolding after heat treatment (Figure 3D). The results showed that both affinity-matured HER3-binders maintained the alpha-helical content and completely refolded after heating to 90°C, as shown by the overlap between spectra generated before and after heating.

### Biosensor Affinity Determination

The equilibrium dissociation constant (K_D_) of the affinity-matured Affibody molecules was determined using biosensor technology. A dilution series of each binder was injected over immobilized human HER3-Fc and the rate constants of the interactions were determined by non-linear regression using a one-site binding model and used to calculate the K_D_ (data not shown). The obtained affinities were estimated to 50 and 21 pM for Z_08698_ and Z_08699_, respectively (Table 1), representing approximately a 33-fold affinity improvement of the strongest binder Z_08699_ compared to Z_05417_. However, due to that the observed on-rates (8.3 • 10^5^ and 1.9 • 10^6^ M^−1^s^−1^, respectively) and off-rates (4.1 • 10^−5^ and 3.9 • 10^−5^ s^−1^, respectively) of Z_08698_ and Z_08699_ were close to the limit of accurate determination of the biosensor instrument, these values should be regarded as approximations. Still, these results are encouraging, demonstrating that the staphylococcal display system indeed is a powerful method for isolation of affinity molecules of low picomolar affinities.

**Table 1:** Affinities of HER3-specific Affibody molecules determined by SPR.

	K_D_ (pM, mean ± SD)^a^	k_a_ (M^−1^s^−1^, mean)^a^	k_d_ (s^−1^, mean)^a^
Z_08698_	50±5.0	8.3×10^5^	4.1×10^−5^
Z_08699_	21±2.4	1.9×10^6^	3.9×10^−5^

a Analyzed in duplicates.

### Labeling and *in vitro* Cell Binding of ^99m^Tc(CO)_3_-Z_08698_-H_6_ and ^99m^Tc(CO)_3_-Z_08699_-H_6_

**Figure 4:**
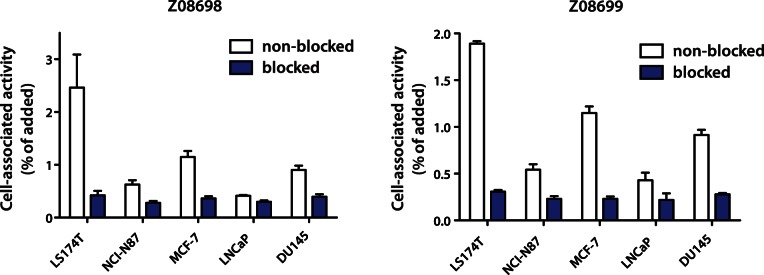
Specificity of binding of labeled Affibody molecules to HER3-expressing cells ***in vitro*** **.** Cells were incubated with 1 nM of radiolabeled conjugates for 1 h. For pre-saturation (blocking) of antigens, 0.7 µM unlabeled non-radioactive Affibody molecules was added. Data are presented as mean values of percent of added radioactivity that is cell-bound from three cell dishes and standard deviations. The difference between uptake by non-blocked and blocked cells was statistically significant (p<0.05).

To confirm that the new binders could target the HER3 receptor on cancer cell lines, the Affibody molecules were radiolabeled using ^99m^Tc(CO)_3_ on the His-tag. The radiolabeling provided a yield of 43±6% for ^99m^Tc(CO)_3_-Z_08698_-H_6_ and 73±12% for ^99m^Tc(CO)_3_-Z_08699_-H_6_. Purification using disposable size-exclusion chromatography NAP-5 columns provided purity of 98.1±0.1% for ^99m^Tc(CO)_3_-Z_08698_-H_6_ and 99±1% for ^99m^Tc(CO)_3_-Z_08699_-H_6_. Binding specificity tests were performed to assess if the binding of ^99m^Tc(CO)_3_-Z_08698_-H_6_ and ^99m^Tc(CO)_3_-Z_08699_-H_6_ to HER3-expressing cells was receptor-mediated. Saturation of the receptors by pre-incubation with non-labeled Affibody molecules significantly (p<0.05) decreased the binding of the radiolabeled Affibody molecule, which suggested that the binding was specific (Figure 4).

### *In vivo* Studies

The biodistribution of the two ^99m^Tc -labeled Affibody molecules at 4 h after injection in female NMRI mice is presented in Table 2. Both conjugates were rapidly cleared from blood and non-HER3-expressing tissues, such as bone and muscle. A high accumulation in kidneys indicated predominately renal excretion of the Affibody molecules. There was a significant difference in uptake of both Affibody molecules in HER3-expressing tissues (i.e. liver, stomach, small intestines and salivary gland) after injection of relatively low (1 µg, 0.13 nmol) compared with high (10 µg, 1.3 nmol) amount of the proteins. The radioactivity uptake in these tissues was lower after injection of higher protein amount (more unlabeled Affibody molecule relative to radiolabeled), suggesting that uptake was saturable and HER3-mediated. The concentration of radioactivity in blood was also lower after injection of the larger protein doses. In both cases, there was increase of renal radioactivity after injection of 10 µg of Affibody molecules. There were observable difference in biodistribution between ^99m^Tc(CO)_3_-Z_08698_-H_6_ and ^99m^Tc(CO)_3_-Z_08699_-H_6_ after injection of 1 µg. The blood concentration and uptake in lung, stomach, kidneys and muscle were significantly lower, but uptake in small intestines was significantly higher in the case of ^99m^Tc(CO)_3_-Z_08698_-H_6_. At an injected dose of 10 µg, the only difference between two conjugates was a lower uptake of ^99m^Tc(CO)_3_-Z_08698_-H_6_ in uterus.

**Table 2:** Biodistribution of ^99m^Tc(CO)_3_-Z_08698_-H_6_ and ^99m^Tc(CO)_3_-Z_08699_-H_6_ in female NMRI mice 4 h after injection.

	^99m^Tc(CO)_3_-Z08698-H_6_		^99m^Tc(CO)_3_-Z08699-H_6_	
	1 µg	10 µg	1 µg	10 µg
blood	0.8±0.1a, c	0.60±0.05	1.1±0.1^b^	0.6±0.1
lung	2.3±0.4^c^	2.3±0.7	3.4±0.3^b^	2.6±0.3
liver	12±2^a^	7.8±0.7	11±2^b^	6.6±1.0
spleen	1.4±0.3	1.7±0.3	1.4±0.2	1.2±0.2
stomach	4.0±0.5^a^	2.2±0.2	4.6±0.2^b^	2.6±0.4
sm intest	8.6±0.6a, c	2.4±0.1	7.7±0.5^b^	2.7±0.5
kidney	85±17a, c	146±16	131±8^b^	163±25
uterus	0.9±0.1a, c	1.2±0.1^d^	1.5±0.1	1.6±0.3
saliv gland	2.3±0.2^a^	1.1±0.3	2.9±0.5^b^	1.4±0.1
muscle	0.33±0.05^c^	0.33±0.09	0.44±0.06	0.38±0.04
bone	0.8±0.2	0.9±0.1	0.9±0.1	0.87±0.06
carcass*	10.5±0.7^c^	10.7±1.0	12.7±1.6	11.1±0.9

Data are expressed as per cent of injected activity per gram and presented as an average (n = 4) and standard deviation.

* Data for carcass are presented as per cent of injected activity per whole sample.

a significant difference (p<0.05) between uptake after injection of 1 and 10 µg of ^99m^Tc(CO)_3_-Z_08698_-H_6_;

b significant difference (p<0.05) between uptake after injection of 1 and 10 µg of ^99m^Tc(CO)_3_-Z_08699_-H_6_;

c significant difference (p<0.05) between uptake after injection of 1 µg of ^99m^Tc(CO)_3_-Z_08698_-H_6_ and ^99m^Tc(CO)_3_-Z_08699_-H_6_;

d significant difference (p<0.05) between uptake after injection of 10 µg of ^99m^Tc(CO)_3_-Z_08698_-H_6_ and ^99m^Tc(CO)_3_-Z_08699_-H_6_;

The data concerning biodistribution of ^99m^Tc(CO)_3_-Z_08699_-H_6_ in nude mice bearing LNCaP xenografts are presented in Table 3. In agreement with data for NMRI mice, increase of protein dose caused a reduction of the uptake in blood, liver and salivary gland. The tumor uptake did not change significantly, although there was a tendency to increased uptake with the higher protein dose. Increase of the protein dose resulted in significantly higher tumor-to-blood, tumor-to-salivary gland, tumor-to-liver and tumor-to-bone ratios.

**Table 3:** Biodistribution of ^99m^Tc(CO)_3_-Z_08699_-H_6_ in male BALB/C nu/nu mice bearing LNCaP prostate cancer xenografts at 6 h after injection.

	Uptake		Tumor-to-organ ratio	
	0.1 µg	1 µg	0.1 µg	1 µg
blood	0.77±0.04^a^	0.63±0.03	2.4±0.7^a^	4.7±1.3
salivary gland	9.3±2.3^a^	5.2±0.4	0.20±0.01^a^	0.56±0.15
lung	1.82±0.09	2.1±0.3	1.0±0.3	1.4±0.2
liver	16.8±1.1^a^	11.9±0.9	0.11±0.03^a^	0.25±0.06
spleen	1.5±0.2	1.4±0.6	1.2±0.3	2.2±0.8
stomach	5.4±0.9	4.1±0.2	0.4±0.1	0.7±0.2
small intestines	10±3	12±3	0.17±0.03	0.24±0.04
kidney	77±9^a^	130±24	0.02±0.01	0.02±0.01
tumor	1.9±0.8	2.9±0.8		
muscle	0.23±0.02	0.26±0.05	8.0±2.0	11±2
bone	0.6±0.1	0.6±0.2	2.8±0.9^a^	5.5**±**1.4
carcass*	10.9±0.3^a^	13±1		

Data are expressed as per cent of injected activity per gram and presented as an average (n = 4) and standard deviation.

* Data for carcass are presented as per cent of injected activity per whole sample.

a Significant difference between biodistribution after injection of 0.1 and 1 µg of ^99m^Tc(CO)_3_-Z_08699_-H_6_ per mouse.

### Inhibition of Cell Proliferation and Receptor Phosphorylation

**Figure 5:**
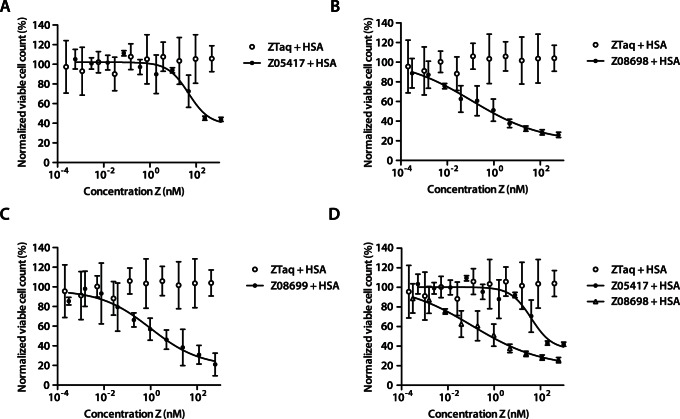
Inhibition of heregulin-induced cell proliferation. MCF-7 cells were treated with heregulin, HSA and a titration series of respective Affibody molecule. Plots showing the normalized viable cell concentration (measured using a CCK-8 kit) on the y-axis and the Affibody concentration on the x-axis. A. Z_05417_ and negative control Z_Taq_. B. Z_08698_ and negative control Z_Taq_. C. Z_08699_ and negative control Z_Taq_. D. Comparison of Z_05417_ and Z_08698_. The signals corresponding to the viable cell concentrations were normalized with the average of the signals from samples treated with the negative control Affibody molecule (Z_Taq_). The experiment was performed in duplicate on different days using freshly prepared solutions and each sample was analyzed at least in triplicate in each experiment. Data is represented as means with indicated standard deviations.

The half maximal inhibitory concentration (IC50) of the Affibody molecules in fusion to an affinity-matured and deimmunized albumin-binding domain (ABD) (based on ABD_035_ for future potential *in vivo* half-life extension [31]) was determined in a heregulin-induced proliferation assay. A dilution series of each binder was mixed with a fixed concentration of heregulin and incubated with MCF-7 cells for five days, both in presence or absence of HSA, respectively. After incubation, viable cell concentration was measured using a colorimetric assay. The IC50 values were determined by non-linear regression of the data to a dose-response equation. In presence of HSA, the obtained IC50 values for Z_08698_ and Z_08699_ were around 0.15 nM and 1.2 nM, respectively, representing an improvement in growth inhibition efficiency of approximately 280-fold and 34-fold, respectively, compared to Z_05417_ (Table 4, Figure 5). Without HSA, the corresponding IC50 values were 0.9 nM and 1.2 nM for Z_08698_ and Z_08699_, respectively (Table 4, data not shown). To investigate potential improvements on inhibition of receptor signaling, an *in vitro* phosphorylation assay was conducted. The affinity-matured binders, Z_08698_ and Z_08699_, demonstrated a concentration dependent inhibition of receptor phosphorylation and a 90% inhibitory effect was obtained with the highest concentration (data not shown). There was hence an approximately 20-fold improvement in the blocking capacity with the affinity-maturated binders compared to the previously obtained data [15] for Z_05416_ and Z_05417_.

**Table 4:** IC50 values of HER3-specific Affibody molecules.

	*With HSA* IC50 (nM, mean ± SD)^a^	*Without HSA* IC50 (nM, mean ± SD)^a^	
Z_08698_	0.15±0.1	0.9±0.6	
Z_08699_	1.2±0.9	1.2±0.3	
Z_05417_	41±20	24±10	

a Mean and SD from duplicate experiments performed on different days. Each experiment was performed with triplicate samples for each concentration.

## Discussion

We believe that the small size of the Affibody molecules (approximately 6.5 kDa) have the potential to provide beneficial tumor targeting attributes, such as rapid extravasation from the blood into the tumor as well as a high degree of diffusivity throughout the tumor. Based on previously isolated Affibody molecules targeting and inhibiting HER3, we set out to generate improved binders as alternatives to conventional targeting molecules like monoclonal antibodies.

A combined combinatorial and rational approach was chosen for the engineering of improved HER3-binders. In order to guide the library design, we started with an alanine scan of the HER3-binding residues in the Affibody molecule, Z_05416_. Alanine scanning using staphylococcal cell surface display allows for rapid and detailed mapping of contributing residues without the need for protein purification. Interestingly, the alanine scanning results revealed the significance of particularly two residues for HER3 binding; a tyrosine at position 10 and a tryptophan at position 17, both located in the first helix of the Affibody molecule and present in both Z_05416_ and Z_05417_ (Figure 1A). Based on the results from the alanine scanning, an affinity maturation library was designed. The library was based on a new optimized Affibody scaffold, containing eleven substitutions in the non-binding region for increased stability and improved attributes for chemical synthesis [27]. In the design of the new affinity maturation library, the mutation frequency in each position was normalized with the alanine scan data, i.e. with its relative importance for HER3-binding. The randomizations were hence designed to yield a relatively high degree of mutations at positions with low significance for binding, and relatively low degree of mutations at positions with high significance (Figure 1B). In average, a mutation frequency of approximately 3 out of 13 positions was employed. The intention was to maintain a relatively high HER3-binding functionality in the library and rely on the previously reported fine-affinity discrimination capacity of the staphylococcal display platform for selection of improved variants [14], [25].

The library was sorted in four rounds against HER3 (Figure 2). To further facilitate the isolation of HER3-specific Affibody molecules with improved tumor-retention capacities, off-rate selection procedures were implemented in the later sorting rounds. After four rounds of sorting, the library was enriched for HER3-binders with improved binding capacity to the receptor compared to the previously best-performing Affibody molecule (Figure 2). The equilibrium dissociation constants of the two binders with highest affinity (Z_08698_ and Z_08699_) were around 50 and 21 pM, respectively, and are among the higher reported affinities of targeting molecules towards the HER3 receptor.

In addition, the new HER3-specific Affibody molecules demonstrated high thermal stability and were able to refold into their alpha-helical structure after heat-induced denaturation, as demonstrated by biosensor analysis and CD spectroscopy (Figure 3). This property should enable efficient labeling of the binders with radionuclides for potential molecular imaging as well as radiotherapy.

As expected, retained receptor-specific binding to the HER3-receptor on various cancer cell lines *in vitro* could be shown by measuring the cellular uptake of radionuclide-labeled binders (Figure 4). For site-specific radiolabeling, chelation of ^99m^Tc(CO)_3_^+^ by the hexahistidine tags was used [32]. Moreover, we utilized the cross-reactivity with murine HER3 to determine if the high-affinity Affibody molecules could target HER3 also *in vivo*. Saturable uptake in murine tissue with normal physiological HER3 expression (i.e. lung, liver, stomach, small intestines, and salivary gland) should be an initial evidence for molecular recognition *in vivo*. Earlier, this approach has been used to show *in vivo* specificity of somatostatin receptor- and EGFR-targeting peptides [33], [34], [35], [36].

^99m^Tc(CO)_3_^+^-conjugation at the hexahistidine tag of the Affibody molecules provides uniformly labeled Affibody molecules with reproducible *in vivo* biodistribution, although the hexahistidine tag has been demonstrated to result in elevated liver uptake due to elevated lipophilicity [30]. *In vivo* biodistribution experiments showed that uptake of both ^99m^Tc(CO)_3_-Z_08698_-H_6_ and ^99m^Tc(CO)_3_-Z_08699_-H_6_ in lung, liver, stomach, small intestines, salivary gland was lower at higher injected protein dose (i.e. reduced specific activity), which is an indication of the saturability of such uptake. This is a strong indication of specific targeting of HER3 by both ^99m^Tc(CO)_3_-Z_08698_-H_6_ and ^99m^Tc(CO)_3_-Z_08699_-H_6_ *in vivo*.

Radionuclide imaging of molecular targets *in vivo* might be a powerful tool for patient stratification for targeted therapy [37]. However, imaging might be challenging if a molecular target is expressed not only in tumors but also in normal tissues. This can appreciably reduce imaging contrast. A combination of a moderate level of overexpression, as in the case of HER3, and expression in normal tissues might make imaging impossible. However, we have shown in earlier studies that increasing the injected protein dose (reduction of specific activity) to an optimal level may suppress radioactivity uptake in normal organs due to saturation of receptor with unlabeled Affibody molecule, but not in tumors [38], [39]. The results from the biodistribution study in tumor-bearing mice suggested that imaging of HER3 expression in prostate cancer using radiolabeled Z_08699_-H_6_ is feasible. The use of injected dose of 1 µg per mouse permitted to increase tumor-to-blood and tumor-to-bone ratios from 2.4±0.7 to 4.7±1.3, and from 2.8±0.9 to 5.5**±**1.4, respectively in comparison with injected dose of 0.1 µg. Taking into account that the major metastatic site of prostate cancer is skeleton, such level of contrast is compatible with *in vivo* imaging. Optimization of labeling chemistry, particularly use of more hydrophobic chelators, would probably decrease liver uptake, making imaging of liver metastases possible as well.

Finally, we explored the therapeutic potential of the affinity-matured Affibody molecules in *in vitro* cell assays. The candidates demonstrated an improved inhibition of ligand-induced HER3 phosphorylation and a substantially higher efficiency in an *in vitro* proliferation assay with low to subnanomolar IC50 values (Figure 5). We believe that the improved affinity of the HER3-binders as well as the demonstrated receptor-specific uptake of the Affibody molecules in HER3-expressing organs in mice are valuable attributes for successful tumor uptake and retention, enabling potential treatment of human cancers dependent on functional HER3-signaling using Z_08698_ and Z_08699_. Altogether, these results encourage further studies of tumor targeting, evaluation of the possible therapeutic effects as well as the potential for imaging of these high affinity Affibody molecules *in vivo*.

## Supporting Information

Figure S1 **Theoretical binomial distribution of mutation frequencies in the library and theoretical calculations on combinatorics and library coverage.** A. Distribution of mutation frequencies in a library that is randomized in 13 positions with a mutation frequency of 23% in each position (3/13), assuming that the incorporation of codons follow a binomial distribution. B. Theoretical calculation of the number of combinations of 3 mutations per molecule in a library design that is intended to randomize 13 positions with 16 different codons and a mutation frequency of 23% in each position (3/13).
(PDF)
