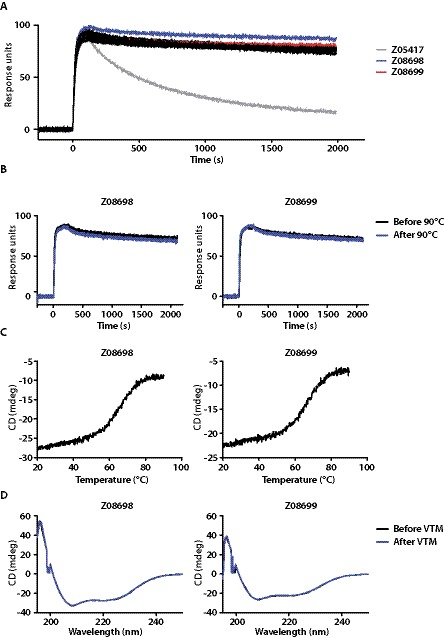# Correction: Inhibiting HER3-Mediated Tumor Cell Growth with Affibody Molecules Engineered to Low Picomolar Affinity by Position-Directed Error-Prone PCR-Like Diversification

**DOI:** 10.1371/annotation/0d29f87c-8dfb-4694-abc7-7284a970910d

**Published:** 2013-11-19

**Authors:** Magdalena Malm, Nina Kronqvist, Hanna Lindberg, Lindvi Gudmundsdotter, Tarek Bass, Fredrik Y. Frejd, Ingmarie Höidén-Guthenberg, Zohreh Varasteh, Anna Orlova, Vladimir Tolmachev, Stefan Ståhl, John Löfblom

Errors were introduced during the preparation of this article for publication. In Figure 3, the x and y axes values have shifted and no longer align with the axes. In the legends for 3A, B, and D the texts have shifted and no longer align with the colored bars. The correct Figure 3 can be viewed here: 

**Figure:**