# Supplementary material for: Inhibiting HER3-Mediated Tumor Cell Growth with Affibody Molecules Engineered to Low Picomolar Affinity by Position-Directed Error-Prone PCR-Like Diversification
Source: PLoS One. 2013 May 10;8(5):e62791. doi: 10.1371/journal.pone.0062791 (PMC3651084; doi:10.1371/journal.pone.0062791)
Supplement: Figure S1 — Theoretical binomial distribution of mutation frequencies in the library and theoretical calculations on combinatorics and library coverage. A. Distribution of mutation frequencies in a library that is randomized in 13 positions with a mutation frequency of 23% in each position (3/13), assuming that the incorporation of codons follow a binomial distribution. B. Theoretical calculation of the number of combinations of 3 mutations per molecule in a library design that is intended to randomize 13 positions with 16 different codons and a mutation frequency of 23% in each position (3/13). (PDF) [file pone.0062791.s001.pdf]

Inhibiting HER3-mediated tumor cell growth with Affibody molecules engineered to low picomolar affinity by position-directed error-prone PCR-like diversification;
PLoS One; Magdalena Malm, Nina Kronqvist, Hanna Lindberg, Lindvi Gudmundsdotter, Tarek Bass, Fredrik Y Frejd,
Ingmarie Höidén-Guthenberg, Zohreh Varasteh, Anna Orlova, Vladimir Tolmachev, Stefan Ståhl and John Löfblom
KTH Royal Institute of Technology, Stockholm, Sweden. E-mail: lofblom@kth.se

Supporting information 1

A

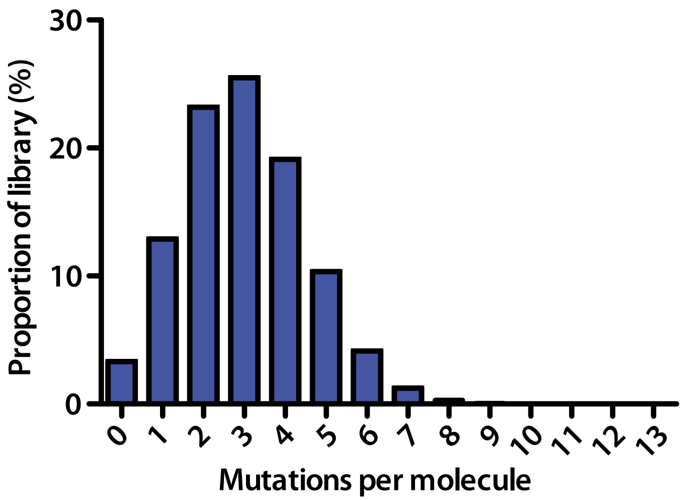

B

P = Number of randomized positions
R = Number of different possible codons in the randomized positions
N = Mutations per molecule
C = Number of possible combinations of N mutations per molecule

$$C = R^N \cdot \text{binomial coefficient} \binom{P}{N} = R^N \cdot \frac{P!}{(P-N)! \cdot N!}$$

Combinations of 3 mutations per Affibody molecule with 13 randomized positions

$$C = R^N \cdot \frac{P!}{(P-N)! \cdot N!} = 16^3 \cdot \frac{13!}{(13\ -3)! \cdot 3!} = 1\ 171\ 456$$

Proportion of Affibody library containing 3 mutations per molecule (see Online Resource 1a) = 25.5 %

$$\text{Library size to cover all combinations of 3 mutations per molecule} = \frac{1\ 171\ 456}{0.255} = 4\ 593\ 945 = 4.6 \cdot 10^6$$

Online Resource 1. Theoretical binomial distribution of mutation frequencies in the library and theoretical calculations on combinatorics and library coverage. a Distribution of mutation frequencies in a library that is randomized in 13 positions with a mutation frequency of 23 % in each position (3/13), assuming that the incorporation of codons follow a binomial distribution. **b** Theoretical calculation of the number of combinations of 3 mutations per molecule in a library design that is intended to randomize 13 positions with 16 different codons and a mutation frequency of 23 % in each position (3/13).
